# Combined Analysis of HLA Class II Eplet Mismatch and Tacrolimus Levels for the Prediction of De Novo Donor Specific Antibody Development in Kidney Transplant Recipients

**DOI:** 10.3390/ijms23137357

**Published:** 2022-07-01

**Authors:** Hyeyoung Lee, Ji Won Min, Hyunhye Kang, Hanbi Lee, Sang Hun Eum, Yohan Park, Chul Woo Yang, Byung Ha Chung, Eun-Jee Oh

**Affiliations:** 1 Department of Laboratory Medicine, International St. Mary’s Hospital, College of Medicine, Catholic Kwandong University, Incheon 22711, Korea; shomermaid@catholic.ac.kr; 2 Division of Nephrology, Department of Internal Medicine, Bucheon St. Mary’s Hospital, College of Medicine, The Catholic University of Korea, Bucheon 14647, Korea; blueberi12@gmail.com; 3 Transplantation Research Center, Seoul St. Mary’s Hospital, College of Medicine, The Catholic University of Korea, Seoul 06591, Korea; hanbilee89@gmail.com (H.L.); trickyspot@gmail.com (S.H.E.); nofever38@naver.com (Y.P.); yangch@catholic.ac.kr (C.W.Y.); 4 Department of Laboratory Medicine, Seoul St. Mary’s Hospital, College of Medicine, The Catholic University of Korea, Seoul 06591, Korea; azuresky@hanmail.net; 5 Division of Nephrology, Department of Internal Medicine, Seoul St. Mary’s Hospital, College of Medicine, The Catholic University of Korea, Seoul 06591, Korea; 6 Division of Nephrology, Department of Internal Medicine, Incheon St. Mary’s Hospital, College of Medicine, The Catholic University of Korea, Seoul 06591, Korea; 7 Division of Nephrology, Department of Internal Medicine, Konyang University Hospital, College of Medicine, Konyang University, Daejeon 35365, Korea

**Keywords:** eplet, donor specific antibody, kidney transplantation, tacrolimus trough level

## Abstract

We investigated whether HLA class II eplet mismatch was related to dnDSA development and analyzed its combined impact with tacrolimus levels for kidney transplantation outcomes. A total of 347 kidney transplants were included. HLA Matchmaker was used for the single molecular eplet, total eplet, antibody (Ab)-verified eplet mismatch analyses, and Ab-verified single molecular analysis to identify HLA-DR/DQ molecular thresholds for the risk of dnDSA development. A time-weighted tacrolimus trough level (TAC-C0) of 5 ng/mL and a TAC-C0 time-weighted coefficient variability (TWCV) of 20% were applied to find the combined effects on dnDSA development. A high level of mismatch for single molecular eplet (DQ ≥ 10), total eplet (DQ ≥ 12), Ab-verified eplet (DQ ≥ 4), and Ab-verified single molecular eplet (DQ ≥ 4) significantly correlated with HLA class II dnDSA development. Class II dnDSA developed mostly in patients with low TAC-C0 and high eplet mismatch. In the multivariable analyses, low TAC-C0 and high eplet mismatch showed the highest hazard ratio for the development of dnDSA. No significant combined effect was observed in dnDSA development according to TWCV. In conclusion, the determination of HLA class II eplet mismatch may improve the risk stratification for dnDSA development, especially in conjunction with tacrolimus trough levels.

## 1. Introduction

Kidney transplantation (KT) is an excellent treatment option for the ever-increasing list of end-stage renal disease patients, but there are many hurdles that clinicians and patients need to overcome [1,2]. The delicate balance between allograft rejection and immunosuppressant-associated complications such as infection, nephrotoxicity, and new-onset diabetes, to name but a few, is difficult to maintain. Therefore, the need to tailor immunosuppression according to individual immunologic risks and characteristics has always been emphasized [2].

It is well known that de novo human leukocyte antigens (HLA) donor-specific antibodies (DSA), especially those that develop against HLA class II, are associated with chronic allograft dysfunction [3,4]. Therefore, rigorous analyses of HLA eplets have been carried out to determine the immunogenicity of HLA mismatches, as well as the relationship of the mismatches with the risk of de novo DSA (dnDSA) development [5,6]. Among many algorithms that identify B-cell and T-cell eplets on HLA, the most well-known algorithm for B-cell eplets is the HLA Matchmaker, which recognizes HLA targets at polymorphic amino acid levels [7,8]. IN recent studies, HLA-DQ and -DR eplet mismatches have been shown to be associated with an increased risk of dnDSA development, acute rejection, transplant glomerulopathy, and graft loss [9,10,11].

There are three known methods for identifying eplet mismatch. Early publications were based on the correlation of allograft outcomes with HLA eplet mismatch data that calculated the sum of mismatched eplets within a certain locus or across multiple loci [12]. However, Wiebe et al. developed an alloimmune prognostic biomarker for dnDSA-free survival by quantifying single eplet mismatches in the HLA-DR and HLA-DQ molecules [13]. This single-molecule method specifies a score for each HLA molecule that is mismatched and correlates the mismatch with alloimmune risk categories for dnDSA, antibody-mediated rejection (ABMR), and allograft survival [12,13]. Finally, the last method utilizes the HLA Epitope Registry, which proposes a list of “antibody-verified” eplets that may be associated with higher immunogenicity, in other words, those that will most likely lead to antibody formation [14]. 

Meanwhile, tacrolimus (TAC)-based immunosuppressive therapy is the most common maintenance regimen used in KT that has been associated with improved graft outcomes over the years [15,16]. However, TAC has a narrow therapeutic range; therefore, clinicians need to perform therapeutic drug monitoring (TDM) to guide prescriptions [17]. In general, low TAC- trough level (C0) can be associated with adverse outcomes such as a high incidence of dnDSA, allograft rejection, and also low allograft survival rates [18,19,20]. In addition, when patients showed high fluctuations of TAC-C0, so-called high TAC-intra-patient variability (IPV) calculated using a time-weighted coefficient of variability (TWCV), allograft outcomes can be worse even in patients with an acceptable mean TAC-C0. However, it has not been established whether TAC-C0 or TAC-IPV has an impact on post-transplant outcomes depending on the degree of eplet mismatch in patients with no DSA at baseline, with only a few reports that have been made so far [18]. Based on this background, we analyzed HLA class II eplet mismatches using the above-mentioned methods and investigated whether they were related to dnDSA development. In addition, we analyzed whether TAC-C0 or TAC-IPV has a differential effect on dnDSA development according to eplet mismatch in patients with no DSA at baseline.

## 2. Results

### 2.1. Baseline Characteristics

The baseline patient demographics are shown in Table 1. HLA class II dnDSA for HLA-DRB1, DQB1, and DQA1 was detected in 25 patients (7.2%). ABMR developed in 20/347 (5.8%) of the recipients post-dnDSA development. The mean number of HLA class II antigen mismatch was significantly higher in recipients who later developed HLA class II dnDSA compared with recipients without dnDSA (2.4 ± 0.9 vs. 1.9 ± 1.2, *p* = 0.018). In the total cohort, 285 (82.1%) patients received basiliximab induction, while 62 (17.9%) patients received thymoglobulin induction. The majority of patients (98.6%) received tacrolimus, mycophenolate, and prednisone, while a few received cyclosporine (0.9%) or sirolimus (0.6%) instead of tacrolimus as maintenance immunosuppression. 

### 2.2. Traditional HLA DR/DQ Antigen Mismatch and the Risk of Class II dnDSA Development 

**Figure 1:**
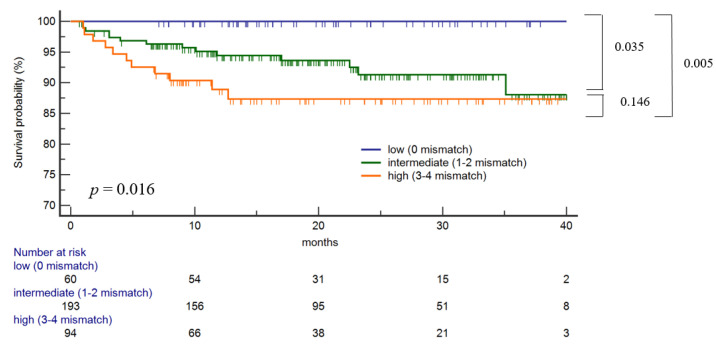
HLA DR/DQ Antigen mismatch on the risk of class II dnDSA development.

HLA antigen mismatches were calculated at the split antigen level. Traditional HLA-DR/DQ antigen mismatch level was associated with dnDSA-free survival (*p* = 0.016) (Figure 1). A low risk of HLA-DR/DQ antigen mismatch (0 mismatches) was associated with significantly lower dnDSA-free survival than intermediate (1–2 mismatches) and high-risk (3–4 mismatches) groups (*p* = 0.035, 0.005, respectively). However, there was no statistical difference in dnDSA-free survival between the intermediate (1–2 mismatches) and high-risk (3–4 mismatches) groups. 

Traditional HLA–DR/DQ antigen mismatch level was associated with dnDSA-free survival (*p* = 0.016). Low risk of HLA-DR/DQ antigen mismatch (0 mismatches) was associated with significantly higher dnDSA-free survival than intermediate (12 mismatches) and high-risk (3–4 mismatches) groups (*p* = 0.035, 0.005, respectively). There was no statistical difference in dnDSA-free survival between the intermediate (1–2 mismatches) and high-risk (3–4 mismatches) groups. 

### 2.3. Defining the Eplet Mismatch Risk Groups

In the single molecular mismatch analysis, the range of HLA-DR eplet mismatch was 0–20, and the range of HLA-DQ was 0–21. We performed eplet mismatch analysis using both HLA alleles and chose the higher value of the mismatch score. We categorized recipients into three alloimmune risk groups as low, intermediate, and high. ROC analysis identified HLA-DR and HLA-DQ single molecular eplet mismatch specific thresholds as >7 for HLA-DR and >9 for HLA-DQ, best associated with dnDSA development with an area under the curve (AUC) of 0.70 and 0.77, respectively. Using this threshold, the recipients were classified into 3 groups: Group A (n = 52) HLA-DR = 0 and HLA-DQ = 0; Group B (n = 72) HLA-DR = 1–7 and HLA-DQ = 1–9; Group C (n = 223) HLA-DR ≥8 or HLA-DQ ≥10. We combined group A and group B into a single low-risk category (HLA-DR ≤ 7 and HLA-DQ ≤ 9) as there was no significant difference in dnDSA development between the two groups, and the sum of the two groups accounted for only 35.7% (124/347) of the total number of patients. We repeated the ROC analysis in the same way for group C patients only. We could not identify additional cutoffs for HLA-DR, but a threshold of HLA-DQ eplet mismatch >9 was detected. We categorized group C recipients into intermediate risk (HLA-DR 8–20 and HLA-DQ ≤ 9) (n = 99), and high risk (HLA-DR 0–20 and HLA-DQ 10–21) (n = 124). 

In the total eplet mismatch analysis, the range of HLA-DR eplet mismatch was 0–45, and the range of HLA-DQ was 0–29. ROC analysis identified HLA-DR and DQ total eplet mismatch specific thresholds as HLA-DR > 13 and HLA-DQ > 9 to be best associated with dnDSA development with an AUC of 0.59 and 0.73, respectively. Using this threshold, the recipients were classified into 3 groups: Group A (n = 52) HLA-DR = 0 and HLA-DQ = 0; Group B (n = 72) HLA-DR = 1–13 and HLA-DQ = 1–9; Group C (n = 223) HLA-DR ≥ 14 or HLA-DQ ≥ 10. Once again, we combined groups A and B into a low-risk category and repeated the ROC curve analysis for only group C patients. There were no cutoffs identified for the HLA-DR locus, but a threshold of HLA-DQ eplet mismatch>11 was detected. Finally, we categorized recipients as low risk (HLA-DR ≤ 13 and HLA-DQ ≤ 11) (n = 124), intermediate risk (HLA-DR 14-45 and HLA-DQ ≤ 11) (n = 98), and high risk (HLA-DR 0–45 and HLA-DQ 12–29) (n = 125). 

In the antibody-verified eplet mismatch analysis, the range of HLA-DR eplet mismatch was 0–14, and the range of HLA-DQ was 0–11. The threshold of HLA-DR > 2 and HLA-DQ > 1 was best associated with dnDSA development with AUCs of 0.61 and 0.72, respectively. After repeated ROC analysis for group C recipients, an additional cut-off for HLA-DQ > 3 was identified. In this group, the recipients were categorized as low risk (HLA-DR ≤ 2 and HLA-DQ ≤ 1) (n = 95), intermediate risk [(HLA-DR ≤ 14 and HLA-DQ 2–3) or (HLA-DR 3-14 and HLA-DQ ≤ 1)] (n = 168), and high risk (HLA-DR 0–14 and HLA-DQ 4–11) (n = 84). 

**Figure 2:**
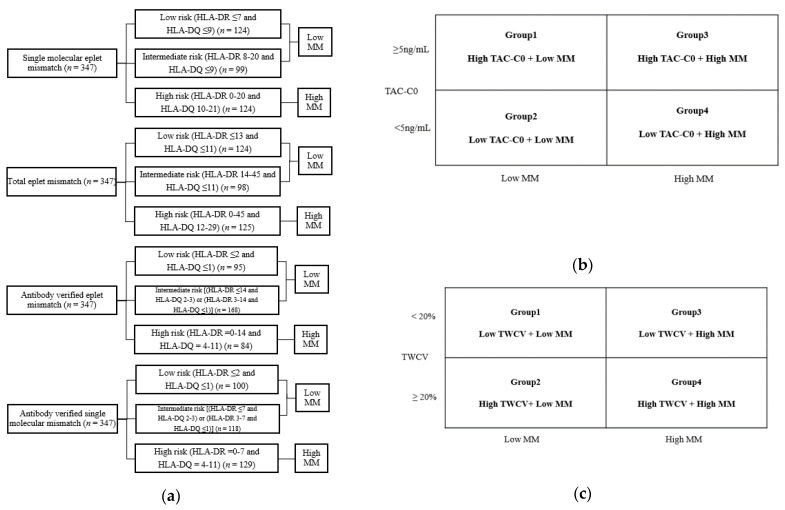
Distribution of patients according to eplet mismatch, TAC-C0 and TAC-IPV. (**a**) Patient distribution according to each eplet mismatch analysis, (**b**) patient groups according to eplet mismatch and TAC-C0, (**c**) patient groups according to eplet mismatch and TAC-IPV. TAC-T0, time-weighted tacrolimus trough level; TAC-IPV, tacrolimus intrapatient variability; TWCV, TAC-C0 time-weighted coefficient variability.

In the antibody-verified single molecular mismatch analysis, the range of HLA-DR eplet mismatch was 0–7, and the range of HLA-DQ was 0–11. The threshold of HLA-DR > 2 and HLA-DQ > 1 was best associated with dnDSA development with AUCs of 0.68 and 0.73, respectively. After repeated ROC analysis for only group C patients, an additional cut-off for HLA-DQ > 3 was identified. In this group, the recipients were categorized as low risk (HLA-DR ≤ 2 and HLA-DQ ≤ 1) (n = 100), intermediate risk [(HLA-DR ≤ 7 and HLA-DQ 2–3) or ((HLA-DR 3–7 and HLA-DQ ≤ 1)] (n = 118), and high risk (HLA-DR 0–7 and HLA-DQ 4–11) (n = 129). The distribution of patients in the risk groups according to eplet mismatch numbers is shown in Figure 2a. 

### 2.4. Eplet Mismatch and Risk of HLA Class II dnDSA 

**Figure 3:**
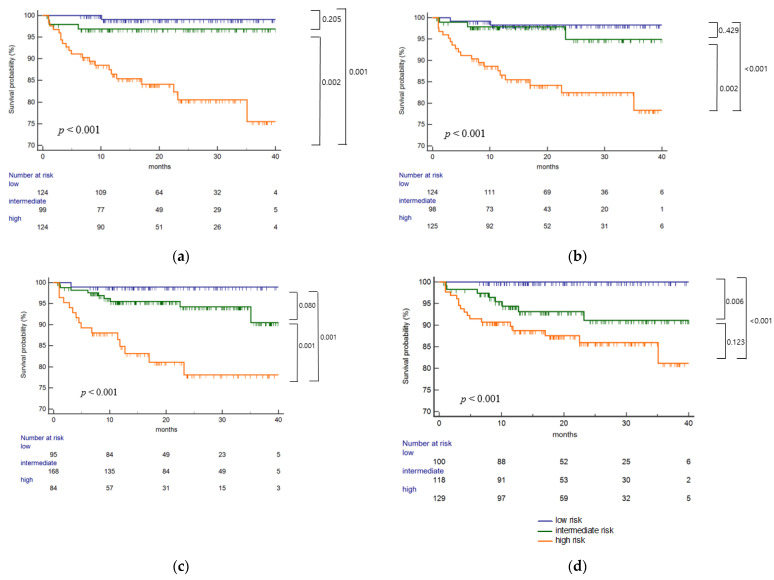
Eplet mismatch analysis on the risk of class II dnDSA development. (**a**) single molecular eplet mismatch, (**b**) total eplet mismatch, (**c**) antibody-verified eplet mismatch and (**d**) antibody-verified single molecular eplet mismatch was significantly associated with the risk of class II dnDSA development (*p* < 0.001, *p* < 0.001, *p* < 0.001, *p* < 0.001, respectively). For single molecular eplet mismatch, the high-risk group (HLA-DQ ≥ 10) showed significantly increased risk of dnDSA development compared to the low (HLA-DR ≤ 7 and HLA-DQ ≤ 9) or intermediate-risk group (HLA-DR ≥ 8 and HLA-DQ ≤ 9) (*p* = 0.001, *p* = 0.002, respectively). For total eplet mismatch, a significant difference was found between the high (HLA-DQ ≥ 12) and intermediate (HLA-DR ≥ 14 and HLA-DQ ≤ 11) risk group (*p* = 0.002) as well as the high and low (HLA-DR ≤13 and HLA-DQ ≤ 11) risk group (*p* < 0.001). For antibody-verified eplet mismatch, the high-risk (DQ ≥ 4) group showed lower dnDSA-free survival compared to the low-risk (HLA-DR ≤ 2 and HLA-DQ ≤ 1) group (*p* = 0.001), with a significant difference between the intermediate [(HLA-DR ≤ 14 and HLA-DQ 2–3) or (HLA-DR 3–14 and HLA-DQ ≤ 1)] and high-risk group (*p* = 0.001). In the antibody-verified single molecular mismatch analysis, the high-risk group (HLA-DQ ≥ 4) showed lower dnDSA-free survival compared to the low-risk (HLA-DR ≤ 2 and HLA-DQ ≤ 1) group (*p* < 0.001), with a significant difference between the low and intermediate [(HLA-DR ≤ 7 and HLA-DQ 2–3) or ((HLA-DR 3–7 and HLA-DQ ≤ 1)] risk group (*p* = 0.006).

In the single-molecular eplet mismatch analysis (Figure 3a), risk categories significantly correlated with HLA class II dnDSA development (*p* < 0.001). The high-risk group showed a significantly increased risk of dnDSA development compared to the intermediate-risk group (*p* = 0.001, *p* = 0.002, respectively), but there was no significant difference between the low- and intermediate-risk groups. Risk categories by total eplet mismatches (Figure 3b) also showed a significant correlation with class II dnDSA development (*p* < 0.001). A significant difference was found between the high- and intermediate-risk groups (*p* = 0.002) as well as the high- and low-risk groups (*p* < 0.001). In the antibody-verified eplet analysis (Figure 3c), the high-risk group showed lower dnDSA-free survival compared to the low-risk group (*p* = 0.001), with a significant difference between the intermediate- and high-risk groups (*p* = 0.001). In the antibody-verified single-molecular mismatch analysis (Figure 3d), the high-risk group showed lower dnDSA-free survival compared to the low-risk group (*p* < 0.001), with a significant difference between the low- and intermediate-risk groups (*p* = 0.006). 

### 2.5. Eplet Mismatch and TAC-C0 on the Risk of HLA Class II dnDSA 

**Figure 4:**
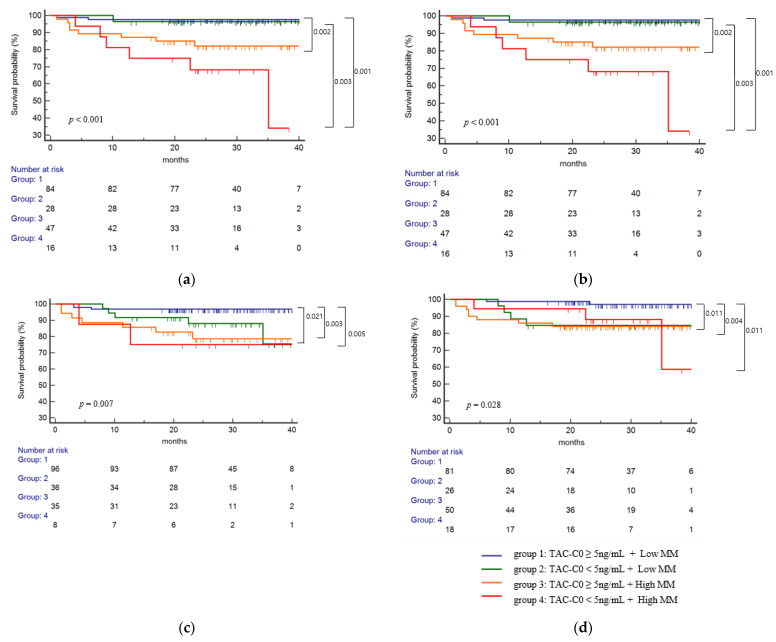
Combined effects of eplet mismatch and TAC-T0 on the risk of class II dnDSA development. Patients were divided into four groups based on time-weighted TAC-C0 up to post-transplant 1st year of 5 ng/mL. (**a**) Combined effects of single molecular eplet mismatch and TAC-T0 for the risk of class II dnDSA development showed worst dnDSA-free survival in group 4 (*p* = 0.007) compared to groups 1 and 2, and worse survival in group 3 compared to group 1, (**b**) total eplet mismatch and TAC-T0 for the risk of class II dnDSA development showed worst dnDSA-free survival in group 4 compared to groups 1 and 2, and worse survival in group 3 compared to group 1, (**c**) antibody-verified eplet mismatch and TAC-T0 for the risk of class II dnDSA development showed highest dnDSA-free survival in group 1 compared to groups 2, 3, and 4, (**d**) antibody-verified single-molecular eplet mismatch and TAC-T0 for the risk of class II dnDSA development showed highest dnDSA-free survival in group 1 compared to groups 2, 3, and 4, (*p* < 0.001, *p* < 0.001, *p* = 0.007, and *p* = 0.028 respectively). TAC-T0, time-weighted tacrolimus trough level; low MM, low and intermediate eplet risk groups combined; high MM, high eplet risk group only.

To observe the combined effects of tacrolimus levels and eplet mismatch on dnDSA formation, patients were divided into four groups based on time-weighted TAC-C0 up to post-transplant 1st year of 5 ng/mL, as shown in Figure 2b. Since there were no significant differences between the low- and intermediate-risk groups for the risk of dnDSA development in the above analyses, the patients were divided into low mismatch (MM) (low- and intermediate-risk groups combined) and high MM (high risk only) groups for this analysis. In the single molecular mismatch analysis (Figure 4a), higher dnDSA-free survival was observed in group 1 compared to group 3 (*p* = 0.002), and the worst dnDSA-free survival was in group 4 compared to groups 1 (*p* < 0.001) and 2 (*p* = 0.003), and a tendency, but not a statistically significant difference was observed compared to group 3 (*p* = 0.119). In the total eplet mismatch analysis (Figure 4b), higher dnDSA-free survival was observed in group 1 compared to group 3 (*p* = 0.008), and the worst dnDSA-free survival was in group 4 compared to groups 1 (*p* < 0.001) and (*p* = 0.011), and a tendency, but not a statistically significant difference was observed compared to group 3 (*p* = 0.175). In the antibody-verified eplet mismatch analysis (Figure 4c), group 2, group 3, and group 4 showed significantly worse dnDSA-free survival compared to group 1 (*p* = 0.021, *p* = 0.001, and *p* = 0.005, respectively). In the antibody-verified single molecular mismatch analysis (Figure 4d), group 2, group 3 and group showed significantly worse dnDSA-free survival compared to group 1 (*p* = 0.011, *p* = 0.004, and *p* = 0.011, respectively). 

### 2.6. Eplet Mismatch and TWCV on the Risk of HLA Class II dnDSA 

**Figure 5:**
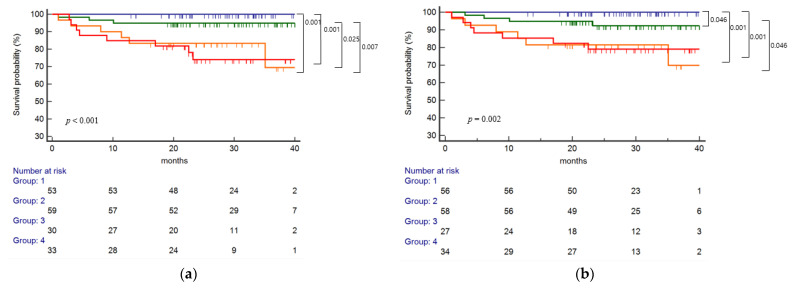

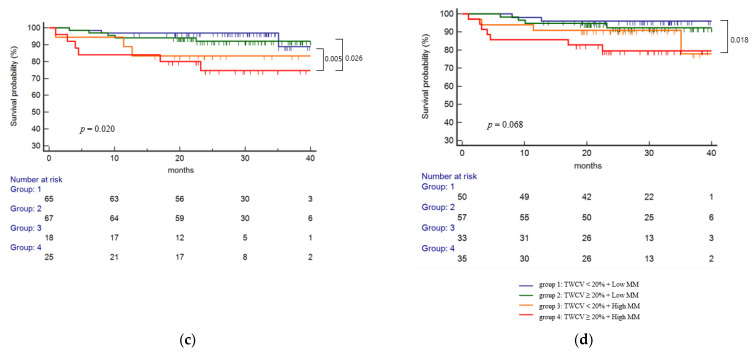
Combined effects of eplet mismatch and TAC-IPV on the risk of class II dnDSA development. (**a**) Combined effects of single molecular eplet mismatch and TAC-IPV (cut off: 20%) for the risk of class II dnDSA development showed higher dnDSA-free survival in group 1 compared to groups 3 and 4, and group 2 compared to groups 3 and 4, (**b**) total eplet mismatch and TAC-IPV for the risk of class II dnDSA development showed higher dnDSA-free survival in group 1 compared to groups 2, 3 and 4, and worse dnDSA-free survival in group 3 compared to group 2, (**c**) antibody-verified eplet mismatch and TAC-IPV for the risk of class II dnDSA development showed higher dnDSA-free survival in group 1 and 2 compared to group 4, (**d**) antibody-verified single molecular eplet mismatch and TAC-IPV for the risk of class II dnDSA development showed higher dnDSA-free survival in group 1 compared to group 4 (*p* < 0.001, *p* < 0.001, *p* = 0.005, *p* = 0.068 respectively). TAC-IPV, tacrolimus intrapatient variability (TAC-IPV); TWCV, time-weighted coefficient of variability; low MM, low and intermediate eplet risk groups combined; high MM, high eplet risk group only.

To further observe the combined effects of TAC levels and eplet mismatch, the patients were divided into four groups according to a TWCV of 20% and eplet mismatch results (Figure 2c). In our study, the median for TWCV distribution was 20.4% (95% CI: 19.2–21.8). In the single molecular mismatch analysis (Figure 5a), higher dnDSA-free survival was observed in group 1 compared to group 3 (*p <* 0.001) and group 4 (*p* < 0.001). Higher dnDSA-free survival was observed in group 2 compared to group 3 (*p =* 0.025) and group 4 (*p* = 0.007). In the total eplet mismatch analysis (Figure 5b), higher dnDSA-free survival was observed in group 1 compared to group 2 (*p =* 0.046), group 3 (*p* < 0.001) and group 4 (*p* < 0.001). Worse dnDSA-free survival was observed in group 3 compared to group 2 (*p* = 0.046). In the antibody-verified eplet mismatch analysis (Figure 5c), higher dnDSA-free survival was observed in group 1 compared to group 4 (*p* = 0.005) and group 2 compared to group 4 (*p* = 0.026). In the antibody-verified single-molecular mismatch analysis (Figure 5d), only group 4 showed worse dnDSA-free survival compared to group (*p* = 0.018). 

### 2.7. Multivariable Analysis for the Risk of HLA Class II dnDSA

Similar results were observed in the multivariable analysis for the risk of HLA class II dnDSA, as shown in Table 2. The models were adjusted for the following characteristics: age, sex, induction type, and ABO compatibility. For groups observing the combined effects of eplet mismatch and TAC-T0 in the single molecular eplet mismatch analysis, the hazard ratio (HR) of groups 3 and 4 compared to group 1 were 9.14 and 16.10, respectively. In the total eplet mismatch analysis, the HR of groups 3 and 4 were 6.99 and 10. In the antibody-verified analysis, the HR of groups 2 and 3 compared to group 1 was 4.69 and 9.51. In the antibody-verified single molecular analysis, the HR of groups 3 and 4 compared to group 1 was 9.14 and 16.10. For groups observing the combined effects of eplet mismatch and TWCV, in the single-eplet mismatch, the HR of groups 3 and 4 compared to group 1 were 13 and 13.41, respectively. In the total eplet mismatch, the HR of groups 3 and 4 were 7.23 and 7.72, and in the antibody-verified eplet mismatch, the HR of groups 3 and 4 compared to group 1 was 4.52 and 4.14. Lastly, in the antibody-verified single molecular eplet mismatch, HR of groups 3 and 4 compared to group 1 was 13.72 and 13.41. The proportional hazard assumptions were validated by the Schoenfeld test (all *p* > 0.05) (Table S1).

### 2.8. Subgroup Analysis for the Risk of HLA-DQ dnDSA 

We performed a subgroup analysis for the risk of HLA-DQ dnDSA in HLA mismatch patients (n = 295). ROC analysis identified HLA-DQ single molecular eplet mismatch and total eplet mismatch specific thresholds as >9 and >11, best associated with HLA-DQ dnDSA development with an AUC of 0.72 and 0.68, respectively. ROC analysis also identified HLA-DQ antibody-verified eplet mismatch and antibody-verified single molecular mismatch specific thresholds as >2 and >2, best associated with HLA-DQ dnDSA development with an AUC of 0.67 and 0.68, respectively. Using this threshold, the recipients were classified into low and high groups for each eplet mismatch analysis (Figure S1). Risk categories significantly correlated with HLA-DQ dnDSA development (*p* < 0.001 for both single molecular eplet mismatch and total eplet mismatch, *p* < 0.05 for both antibody-verified eplet analysis and antibody-verified single molecular mismatch analysis).

We performed an analysis of TAC-C0 levels and eplet mismatch on HLA-DQ dnDSA formation, as shown in Figure S2. The worst HLA-DQ dnDSA-free survival was in group 4 compared to group 1 (*p* < 0.001) and compared to group 2 (*p* = 0.019) in the single molecular mismatch analysis. Furthermore, lower dnDSA-free survival was observed in group 3 compared to group 1 (*p* = 0.012). Higher dnDSA-free survival was observed in group 1 compared to group 3 (*p* = 0.035) and compared to group 4 (*p* = 0.002) in the total eplet mismatch analysis. In the antibody-verified eplet mismatch analysis, there was no statistical significance between groups. In the antibody-verified single molecular mismatch analysis, group 4 showed significantly worse dnDSA-free survival compared to group 1 (*p* = 0.018). 

We performed an analysis of TWCV levels and eplet mismatch on HLA-DQ dnDSA formation, as shown in Figure S3. In the single-molecular mismatch analysis, higher dnDSA-free survival was observed in group 1 compared to group 3 (*p* = 0.007) and group 2 compared to group 4 (*p* = 0.042). Worse dnDSA-free survival was observed in group 4 compared to group 1 (*p* = 0.021), and in group 3 compared to group 2 (*p* = 0.021). In the total eplet mismatch analysis, worse dnDSA-free survival was observed in group 3 compared to group 1 (*p* = 0.018) and group 2 (*p* = 0.028). In the antibody-verified eplet mismatch analysis, lower dnDSA-free survival was observed in group 4 compared to group 2 (*p* = 0.020). In the antibody-verified single-eplet mismatch analysis, there was no statistical significance between groups. 

As seen in Table S2, a multivariable analysis for class II dnDSA was performed using the HLA-DQ subgroup. The groups observing the combined effects of eplet mismatch and TAC-T0 showed some combined effects for risk prediction of HLA class II dnDSA formation in the four analyses. In the single molecular eplet mismatch analysis, the HR of groups 3 and 4 compared to group 1 were 3.77 (95% CI 1.41–10.09, *p* = 0.008) and 8.03 (95% CI 2.62–24.66, *p* = 0.000), respectively. In the total eplet mismatch analysis, the HR of groups 3 and 4 were 3.21 (95% CI 1.18–8.68, *p* = 0.022) and 6.22 (95% CI 2.07–18.66, *p* = 0.001). In the antibody-verified analysis, groups 2 and 3 compared to group 1 showed a HR of 4.85 (95% CI 1.28–18.29, *p* = 0.019), and 3.17 (95% CI 1.18–8.52, *p* = 0.022). In the antibody-verified single molecular analysis, groups 3 and 4 compared to group 1 showed a HR of 3.06 (95% CI 1.13–8.28, *p* = 0.028) and 4.09 (95% CI 1.19–14.00, *p* = 0.025). The groups observing combined eplet mismatch and TWCV showed some combined effects for risk prediction of HLA class II dnDSA in two of the four types of eplet mismatch analysis. In the single-eplet mismatch, the HR of groups 3 and 4 compared to group 1 were 5.17 (95% CI 1.79–14.85, *p* = 0.002) and 4.16 (95% CI 1.39–12.47, *p* = 0.011), respectively. In the total eplet mistmatch, the HR of groups 3 and 4 were 4.56 (95% CI 1.57–13.26, *p* = 0.005) and 3.42 (95% CI 1.17–9.97, *p* = 0.024).

### 2.9. Eplet Mismatch and Antibody Mediated Rejection Development

**Figure 6:**
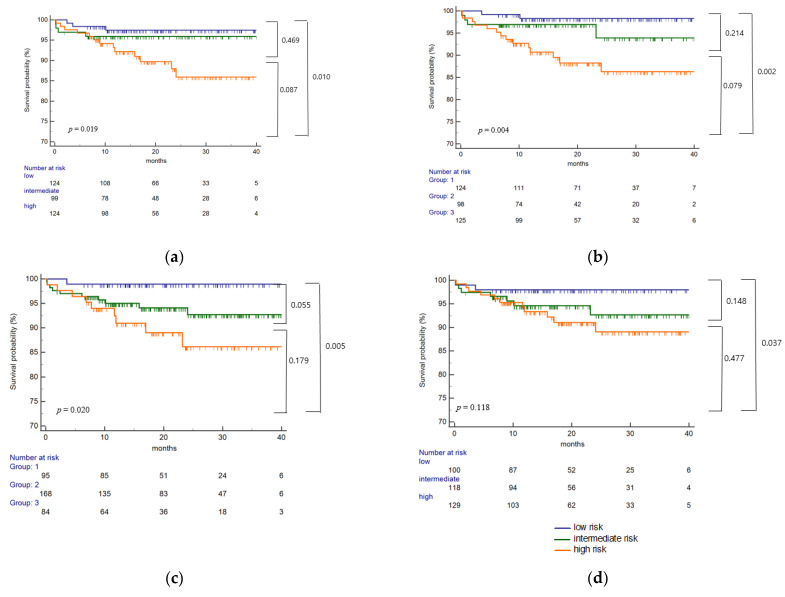
Eplet mismatch on the risk of antibody-mediated rejection development. ABMR-free survival was significantly associated with single molecular mismatch (**a**), total eplet mismatch (**b**), and antibody-verified eplet mismatch (**c**) risk categories (*p* = 0.019, *p* = 0.004, and *p* = 0.020, respectively). The high-risk group showed worse ABMR development compared with low-risk group in single molecular, total eplet mismatch, and antibody verified eplet mismatch (*p* = 0.010, *p* = 0.002, and *p* = 0.005, respectively). In antibody verified single molecular mismatch analysis (**d**), the high-risk group showed worse ABMR development than low-risk patients (*p* = 0.037). ABMR, antibody-mediated rejection.

ABMR-free survival was significantly associated with single molecular (Figure 6a), total eplet mismatch (Figure 6b), and antibody-verified eplet mismatch (Figure 6c) risk categories (*p* = 0.019, *p* = 0.004, and *p* = 0.020, respectively). The high-risk group showed worse ABMR-free survival compared with the low-risk group in single molecular, total eplet mismatch, and antibody-verified eplet mismatch (*p* = 0.010, *p* = 0.002, and *p* = 0.005, respectively). In the antibody-verified single molecular mismatch analysis, the high-risk group showed worse ABMR-free survival than the low-risk patients (*p* = 0.037) (Figure 6d). 

## 3. Discussion

Our knowledge of the clinical significance of different aspects of the HLA molecule is advancing rapidly [21]. Computer algorithms for determining eplet differences, including the HLA Matchmaker, Cambridge HLA Immunogenicity Algorithm, Predicted Indirectly ReCognizable HLA Epitopes (PIRCHE-II) algorithm, and HLA epitope mismatch algorithm (HLA-EMMA), have been developed, and these algorithms are constantly evolving [22,23,24,25]. While early iterations of algorithms determined eplet mismatches between recipients and donors simply based on linear amino acid sequences, more sophisticated tools are being introduced that consider biochemical properties such as electrostatic differences. Despite the differences in algorithms, the degree of eplet mismatch derived from all methods showed a similar correlation with dnDSA formation [26]. 

In this study, we performed eplet mismatch using four different methods, and we confirmed that there was a difference in the occurrence of dnDSA according to the degree of eplet mismatch in all four types of analysis. In addition, effects were shown in predicting the occurrence of dnDSA by combining the results of eplet mismatch analysis with TAC trough levels. We used different cut-off values for eplet mismatch risk compared to those used by Wiebe et al. [13]. The reason for this may be because we did not include HLA DRB3/4/5 typing due to the ethnic variations in HLA typing [27], smaller cohort sizes, and the different version of HLA Matchmaker used for analysis (version 3.1 vs. 2.0). When we applied the same single-molecular cut-offs for HLA-DQ used in Wiebe et al. [13], the risk categories showed a significant correlation with class II dnDSA development (*p* = 0.005) in our cohort as well (Figure S4). The high-risk group (HLA-DQ ≥ 15) showed a significantly increased risk of dnDSA development compared to the low (HLA-DQ ≤8) (*p* < 0.001). There was a significant difference between the low- and intermediate- (HLA-DQ 9–14) risk group (*p* = 0.005), but no significant difference was observed between the intermediate and high-risk group (*p* = 0.263). However, the significance was lost in the tacrolimus trough level and TWCV analysis (data not shown). 

The result of our study is fully consistent with previous studies, which reported that a higher number of eplet mismatches in HLA class II (particularly HLA DR and HLA DQ) increases the risk of HLA class II dnDSA [10,11,28]. In our study, when the recipients were divided by HLA Ag mismatch, the incidence of dnDSA was significantly different between the low risk (HLA Ag mismatch = 0) and intermediate/ high risk (HLA Ag mismatch 1 or higher) groups. However, there was no difference in the incidence of dnDSA between the intermediate group (HLA Ag mismatch 1–2) and the high-risk (Ag mistmach 3–4) group. On the other hand, we observed a difference in the occurrence of dnDSA between the intermediate and the high-risk groups in the eplet mismatch analyses. These findings were observed equally regardless of the eplet mismatch analysis method used. The combined approach of HLA Ag mismatch and eplet mismatch analysis can be anticipated to identify the group of patients with a high risk of developing dnDSA among those with moderate or high levels of HLA Ag mismatch that are difficult to distinguish using the conventional HLA Ag mismatch method.

Our study has shown some advancement in that we confirmed HLA-DR and DQ with high-resolution typing. Many previous papers have limitations concerning the fact that eplet matching was performed using low-resolution HLA typing methods. Eplet analysis of low- or intermediate-resolution typing may present with inaccuracies caused by imputational methods that utilize general-population-based allele and haplotype frequencies [29,30,31,32]. However, since our study performed high-resolution typing on the HLA DRB1, DQA1, and DQB1 locus, the possibility of analytical errors due to imputation can be excluded. It is also significant that we performed eplet analysis using the four types of methods used in B-cell eplet mismatch known so far. According to our results, the four molecular mismatch analyses showed similar results. 

Defining the immunogenicity of eplets is an area that needs to be further evaluated in solid organ transplantation. As not all molecular mismatches lead to antibody formation, knowledge of the immunogenicity of individual HLA eplets is required before eplet matching is implemented in clinical transplantation [33]. Antibody verification is the most basic method for assessing the clinical relevance of an individual eplets by experimenting that the eplet can bind to alloantibodies. Several studies have identified that the subset of antibody-verified eplet mismatches were risk factors for allograft rejection and graft loss [32,34,35]. The immunogenicity can be assessed by large clinical datasets that correlate specific eplet mismatches to DSA formation or poor transplant outcomes. Several studies have investigated, and most studies focus on, the HLA-DQ locus [11,36,37,38]. These different approaches are complementary. Th evaluation of multiple, large patient cohorts would be necessary, and building a large reference dataset of transplant recipients can contribute to the investigation of the predictive values of each eplet type and mismatch score [33]. 

Next, we further analyzed whether eplet mismatches have a combined impact with TAC-C0 or TWCV on the development of dnDSA. Insufficient exposure to immunosuppression is a significant risk factor leading to the development of dnDSA, and previous reports have shown that low TAC-C0 or high TAC-IPV can be associated with the risk of developing dnDSA and other inferior outcomes [39,40]. However, there have been only a few studies analyzing the association between eplet and TAC-C0 [18], and the association with TAC-IPV has not been reported yet. Although previous studies have shown that both eplet mismatch and tacrolimus levels affect the incidence of dnDSA, it has not been established whether TAC-C0 or TAC-IPV has an impact on the post-transplant outcomes depending on the degree of eplet mismatch in patients with no DSA at baseline. We divided the patient groups according to tacrolimus trough levels and the degree of eplet mismatch and confirmed the combined effect of tacrolimus level and eplet mismatch on dnDSA development. In this study, we chose the limits of TAC-T0 5 ng/dL and TWCV 20% based on the results of previous studies that have shown an increased risk of graft failure or poor graft outcomes for patients with a TAC-T0 below 5 ng/dL and TWCV > 20% [17,41,42]. We observed that eplet mismatch load and low TAC-T0 or high TWCV had a combined effect on the occurrence of dnDSA. Not all comparisons were statistically significant, but the tendency for worst dnDSA-free survival was observed in groups with high eplet mismatch and low TAC-T0 or high TWCV, and highest dnDSA-free survival was observed in groups with low eplet mismatch and high TAC-T0 or low TWCV. This tendency was observed similarly in all four types of analysis. Observation of more significant differences may be expected in larger cohort studies with longer follow-up durations.

A limitation of our study is that the follow-up duration is relatively short. Biopsy-proven antibody-mediated rejection was diagnosed in 20 recipients (5.7%), and we only observed the limited association between the development of ABMR and eplet mismatch. It may have been difficult to find the association with chronic ABMR due to the short follow-up period. Additionally, other factors influencing TAC-C0 or TAC-IPV, such as the CYP3A5 genotype, were not considered [43]. Because the target level for tacrolimus frequently changes after transplantation, it is most accurate to compare the risk associated with the level of tacrolimus at each time point. However, this subset analysis cannot be performed in our study due to the small number of events. Risk quantification should be carefully interpreted due to the small sample size and related errors, and risk stratification methods should be prospectively evaluated in a larger independent cohort to confirm their general applicability. We only included four variables in the multivariable analysis to avoid overfitting. Finally, most of the DSA detected in our group was the DQ locus. HLA-DRB1 plays little or no role in patient risk stratification, as only six patients developed DSA against HLA-DR. HLA typing for DRB*3/4/5 was not performed, and this is a major limitation of this study. So, we further analyzed for the subgroup cohort to simplify the models and base them exclusively on DQ DSA prediction. We performed subgroup analysis for the risk of HLA-DQ DSA only in patients with HLA mismatches, as the molecular mismatch algorithms are supposed to predict the risk of DSA development in the presence of an HLA mismatch. We found results similar to that of the total cohort analysis. 

In conclusion, the determination of eplet mismatch load can contribute to improved immunological risk stratification by supplementing HLA antigen mismatch analysis and may help regulate personalized post-transplant follow-up and tailor immunosuppressive therapy. Single molecular eplet mismatch, total eplet mismatch, antibody-verified eplet mismatch, and antibody-verified single-molecular mismatch were each significant correlates of dnDSA development and these effects combined with TAC trough and IPV levels further stratified risk categories. For now, the choice of one method over another would be made according to familiarity and availability, as no evidence of superiority of one method over the others exists. Relevant studies involving larger independent cohorts are needed. 

## 4. Materials and Methods

### 4.1. Study Population

A total of 415 consecutive kidney transplantations performed at Seoul St. Mary’s Hospital from December 2016 to December 2020 were enrolled. Patients with pre-transplant DSA were excluded (n = 68), and a total of 347 patients were included in the final study. The presence of HLA antibodies was analyzed using panel-reactive antibody (PRA) screening and/ or single-antigen bead (SAB) assay. In patients with negative PRA screening results, SAB was not performed, and in patients with positive PRA screening results, the SAB assay was performed. Those with a DSA mean fluorescence intensity (MFI) of >1000 were excluded. The median follow-up period for dnDSA monitoring was 20.3 (95% confidence interval [CI]: 18.5–22.2) months. This study was carried out in accordance with the Declaration of Helsinki and was approved by the Seoul St. Mary’s hospital Institutional Review Board (KC20RIS0767). 

### 4.2. HLA Typing, Antibody Screening and Eplet Analysis

High-resolution HLA typing for DRB1, DQB1, and DQA1 was performed using sequence-specific oligonucleotide probes (One Lambda Inc., A Thermo Fisher Scientific Brand, Canoga Park, CA, USA). HLA typing for DRB*3/4/5 was not performed. The detection of DSA was performed by using LABScreen Single Antigen (One Lambda Inc., A Thermo Fisher Scientific Brand, Canoga Park, CA, USA) as described in previous studies [44,45]. EDTA treatment was used to prevent the prozone effect. An MFI of 1000 was used as a cut-off for positivity. The DSA levels were checked 1 month, 3 months, 6 months, and 12 months post-transplant, and then yearly thereafter. HLA matchmaker software (HLA DRDQDP Matching version 3.1) was used for the single molecular eplet, total eplet, and antibody-verified eplet mismatch analyses. 

### 4.3. Immunosuppression Regimen

Immunosuppression was administered according to the immunosuppressive regimen at our center, as previously described [46]. Briefly, standard immunosuppression consisted of cyclosporine or tacrolimus with mycophenolate mofetil and prednisolone or deflazacort. Induction therapy was performed using either basiliximab (20 mg on postoperative days 0 and 4) or thymoglobulin (15 mg/kg for 5 consecutive days since postoperative day 0) based on the patient’s pre-transplant immunologic risk profile. High-risk patients in living donor kidney transplants such as retransplant patients, high-PRA patients, and ABO-incompatible transplant patients, and in deceased donor kidney transplants, patients receiving kidneys from extended criteria donors received thymoglobulin induction. Desensitization in ABO-incompatible transplant patients was administered as previously reported by our group [47].

### 4.4. Tacrolimus Variability

Tacrolimus levels were measured by Dimension EXL Integrated Chemistry System (Siemens Healthcare Diagnostics, Deerfield, IL, USA) using affinity chrome-mediated immunoassay with trough targets of 8–12 ng/mL within 3 months post-transplant and 5–8 ng/mL thereafter. Tacrolimus levels were checked daily for 2 weeks after kidney transplantation and checked once a week twice, then once every 2 weeks twice, once every 3 weeks twice, every month for 1 year, then every 2 months until 2 years post-transplant. Changes may be made according to the patient’s status. Only the patients with more than 3 tacrolimus measurements available were included in the analysis (n = 175). The mean number of tacrolimus measurements available per patient was 13.80 ± 2.98. To avoid bias, the tacrolimus levels after dnDSA formation were excluded. Time-weighted means, standard deviation, and the coefficient of variation were calculated using previously reported methods [48,49]. Briefly, the time-weighted average of TAC-C0 was calculated as follows: $TW\mu =\frac{1}{t}\sum_{n=1}^{i}xiti$, and the time-weighted standard deviation was calculated as follows: $TW\sigma =\sqrt{\frac{1}{t}\sum_{n=1}^{i}{\left(xi-u\right)}^{2}ti}$. Here, *i* stands for the patient’s outpatient clinic visit number post-transplant, *xi* stands for the TAC-C0 during the time in-between, *t* is the number of days the patient was exposed to the drug, and *ti* is the number of days in the interval time. The variability was estimated by means of the TAC-C0 time-weighted coefficient variability (TWCV), calculated as follows: $\mathrm{TWCV}\left(\%\right)=\left(\mathrm{Time}\;\mathrm{weighted}\;\mathrm{standard}\;\mathrm{deviation}\;/\;\mathrm{time}\;\mathrm{weighted}\;\mathrm{average}\right)\times 100$. To observe the combined effects of tacrolimus levels and eplet mismatch on dnDSA formation, the patients were divided into four groups based on time-weighted TAC-C0 up to post-transplant 1st year (Figure 2b) and TAC-IPV, as shown in Figure 2c.

### 4.5. Statistical Analysis

All analyses were performed using MedCalc^®^ Statistical Software version 20.015 (MedCalc Software Ltd., Ostend, Belgium). The comparisons for continuous variables were performed using the Mann–Whitney test, and comparisons for categorical variables were made using Fisher’s exact test or a chi-square test. We used the two-tailed significance level of *p* < 0.05. ROC analysis with the Youden index was used to identify HLA-DR or HLA-DQ molecular-specific thresholds for the risk of dnDSA development. DSA-free survival analysis was performed using the Kaplan–Meier model, with the log-rank test for significance. Cox proportional-hazards regression was performed for univariate and multivariable analysis to determine the predictors of dnDSA formation. The proportional hazard assumptions were validated by the Schoenfeld test using R software version 3.6.3.

## Figures and Tables

**Table 1:** Baseline patient demographics.

	Total (*n* = 347)	dn Class II DSA (+) (*n* = 25)	dn Class II DSA (−) (*n* = 322)	*p* Value
Age (years) mean ± SD	50.0 ± 12.3	47.4 ± 11.6	50.3 ± 12.4	0.249
Male recipient, *n* (%)	223 (64.3)	16 (64.0)	207 (64.0)	0.998
Deceased donor, *n* (%)	22 (6.3)	1 (4.0)	21 (6.5)	0.619
Retransplantation, *n* (%)	29 (8.4)	1 (4.0)	28 (8.7)	0.703
ABO incompatible, *n* (%)	95 (27.4)	7 (28.0)	88 (27.3)	0.942
High PRA (>50%), *n* (%)	64 (17.1)	3 (12.0)	61 (18.9)	0.389
Preexisting renal disease				0.701
DM, *n* (%)	98 (28.2)	8 (32)	90 (27.9)	
HTN, *n* (%)	47 (13.5)	3 (12.0)	44 (13.7)	
Chronic GN, *n* (%)	119 (34.3)	8 (32.0)	111 (34.5)	
PCKD, *n* (%)	15 (4.3)	0	15 (4.7)	
SLE, *n* (%)	12 (3.5)	2 (8.0)	10 (3.1)	
Others, *n* (%)	56 (16.1)	4 (16.0)	52 (16.1)	
Preemptive KT, *n* (%)	125 (56.1)	10 (40.0)	115 (35.7)	
Follow-up (months), mean ± SD	21.5 ± 10.6	21.5 ± 10.6	21.5 ± 10.6	0.975
HLA class I Ag MM, mean ± SD	3.1 ± 1.7	3.4 ± 1.6	3.1 ± 1.7	0.348
HLA class II Ag MM, mean ± SD	2.0 ± 1.2	2.4 ± 0.9	1.9 ± 1.2	0.018
Induction, *n* (%)				0.801
Basiliximab	285 (82.1)	21 (84.0)	264 (82.0)	
ATG	62 (17.9)	4 (16.0)	58 (18.0)	
Maintenance immunosuppression, *n* (%)				0.821
Tacrolimus	342 (98.6)	25 (100)	317 (98.4)	
Cyclosporine	3 (0.9)	0	3 (0.9)	
Sirolimus	2 (0.6)	0	2 (0.6)	

dn, de-novo; DM, diabetes mellitus; HTN, hypertension; GN, glomerulonephritis; PCKD, polycystic kidney disease; SLE, Systemic lupus erythematosus; KT, kidney transplantation; MM, mismatch; ATG, anti-thymocyte globulin.

**Table 2:** Multivarible analysis for class II dnDSA development (n = 175) ^a^.

	Single Molecular Eplet		Total Eplet		Antibody-Verified Eplet		Antibody-Verified Single Molecular	
	HR (95% CI)	*p* Value	HR (95% CI)	*p* Value	HR (95% CI)	*p* Value	HR (95% CI)	*p* Value
Eplet MM & TAC-T0								
Group 1	Reference		Reference		Reference		Reference	
Group 2	1.51 (0.14–16.67)	0.736	1.14 (0.12–10.92)	0.912	4.69 (1.12–19.64)	0.034	1.51 (0.14–16.67)	0.736
Group 3	9.14 (1.97–42.40)	0.005	6.99 (1.81–26.95)	0.005	9.51 (2.48–36.50)	0.001	9.14 (1.97–42.40)	0.005
Group 4	16.10 (3.12–83.23)	0.001	10.13 (2.41–42.45)	0.002	5.04 (0.52–48.61)	0.162	16.10 (3.12–83.23)	0.001
Eplet MM & TWCV								
Group 1	Reference		Reference		Reference		Reference	
Group 2	1.78 (0.16–19.59)	0.640	0.92 (0.13–6.56)	0.935	0.97 (0.24–3.88)	0.964	1.78 (0.16–19.59)	0.640
Group 3	13.72 (1.69–111.51)	0.014	7.23 (1.46–35.86)	0.016	4.52 (1.12–18.21)	0.034	13.72 (1.69–111.51)	0.014
Group 4	13.41 (1.65–109.21)	0.015	7.72 (1.57–37.94)	0.012	4.14 (1.10–15.55)	0.035	13.41 (1.65–109.21)	0.015

^a^ Models were adjusted for the following characteristics: age, sex, induction type, and ABO compatibility. Univariable results with *p*-value < 0.2 were included in the multivariable analysis. Multivariable analysis of each eplet mismatch category was performed separately. Group 1 represents patients with high TAC-T0 + low/intermediate eplet mismatch, Group 2 represents patients with low TAC-T0 + low/intermediate eplet mismatch, Group 3 represents patients with high TAC-T0 + high eplet mismatch, Group 4 represents patients with low TAC-T0 + high eplet mismatch.

## Data Availability

The data presented in this study are available in article.

## Supplementary Materials

The following supporting information can be downloaded at: https://www.mdpi.com/article/10.3390/ijms23137357/s1.
