# Supplementary material for: Combined Analysis of HLA Class II Eplet Mismatch and Tacrolimus Levels for the Prediction of De Novo Donor Specific Antibody Development in Kidney Transplant Recipients
Source: Int J Mol Sci. 2022 Jul 1;23(13):7357. doi: 10.3390/ijms23137357 (PMC9267119; doi:10.3390/ijms23137357)
Supplement: Supplementary file 1 [file ijms-23-07357-s001.zip › ijms-1767491-supplementary/Supplementary figure.pdf]

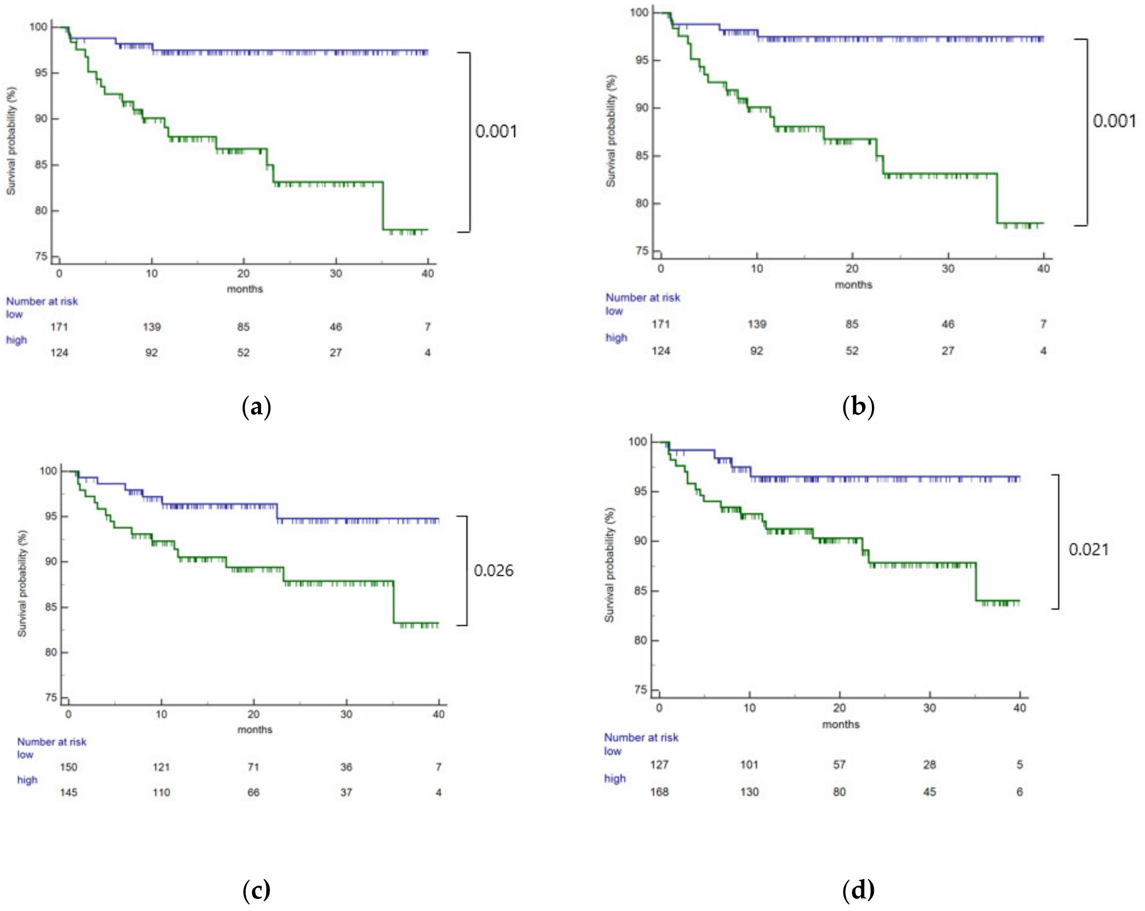

Figure S1. HLA-DQ eplet mismatch analysis on the risk of HLA-DQ dnDSA development in class II HLA mismatched recipients (n=295). The high risk group of (a) single molecular eplet mismatch (HLA-DQ >9), (b) total eplet mismatch (HLA-DQ >11), (c) antibody verified eplet mismatch (HLA-DQ >2) and (d) antibody verified single molecular eplet mismatch (HLA-DQ >2) was significantly associated with the risk of class II dnDSA development ($\rho < 0.001$ for both single molecular eplet mismatch and total eplet mismatch, $\rho < 0.05$ for both antibody verified eplet analysis and antibody verified single molecular mismatch analysis).

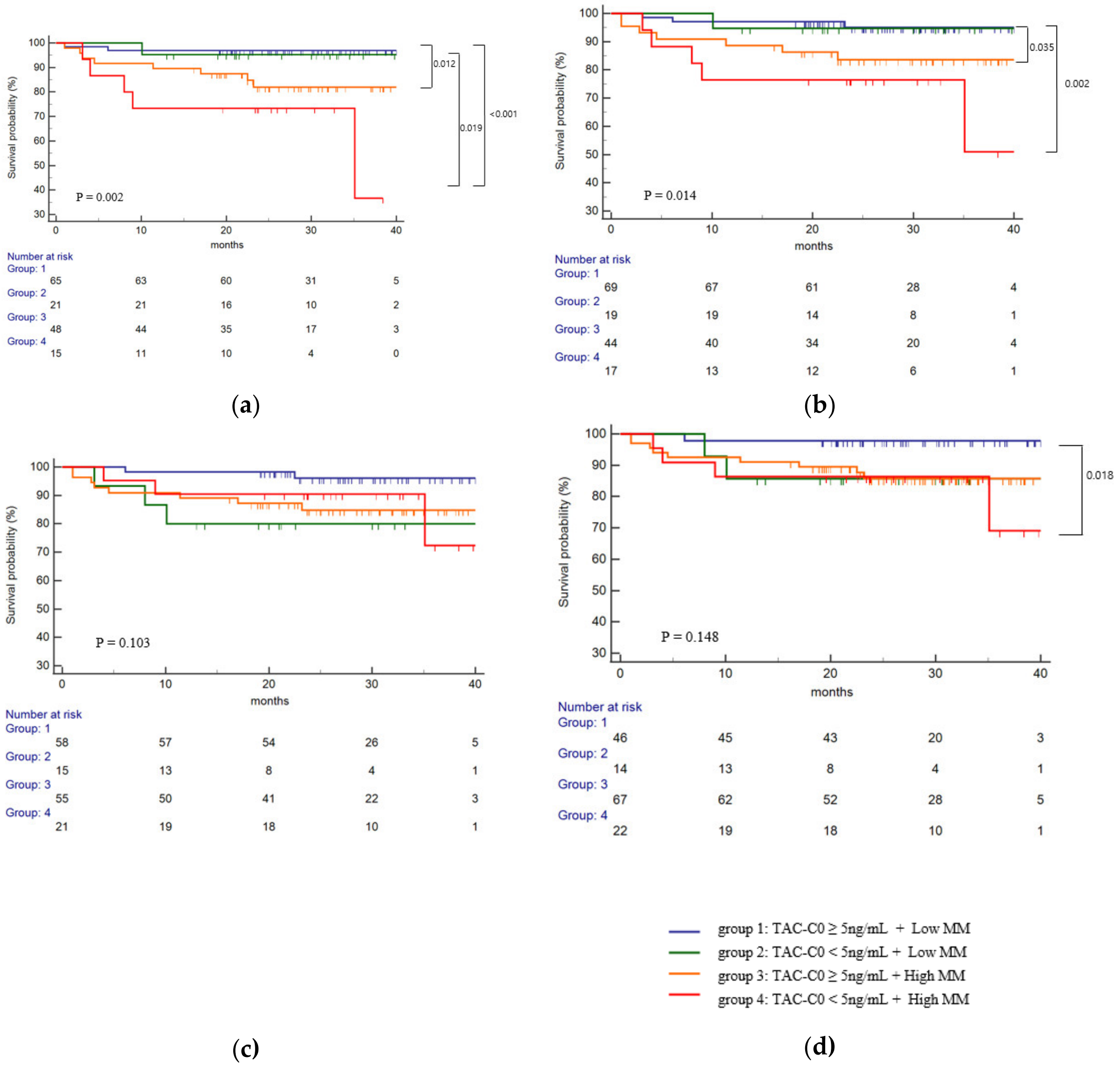

Figure S2. Combined effects of HLA-DQ eplet mismatch and TAC-T0 on the risk of HLA-DQ dnDSA development in class II HLA mismatched recipients. (a) In the single molecular mismatch HLA-DQ dnDSA-free survival was worse in group 4 compared to group 1 ($\rho < 0.001$) and group 2 ($\rho = 0.019$). Lower dnDSA-free survival was observed in group 3 compared to group 1 ($\rho = 0.012$). (b) In total eplet mismatch, higher dnDSA-free survival was observed in group 1 compared to group 3 ($\rho = 0.035$) and group 4 ($\rho = 0.002$). (c) In the antibody verified eplet mismatch analysis, there was no statistical significance between groups. (d) In the antibody verified single molecular mismatch analysis, group 4 showed significantly worse dnDSA-free survival compared to group 1 ($\rho = 0.018$).

* TAC-T0, time-weighted tacrolimus trough level

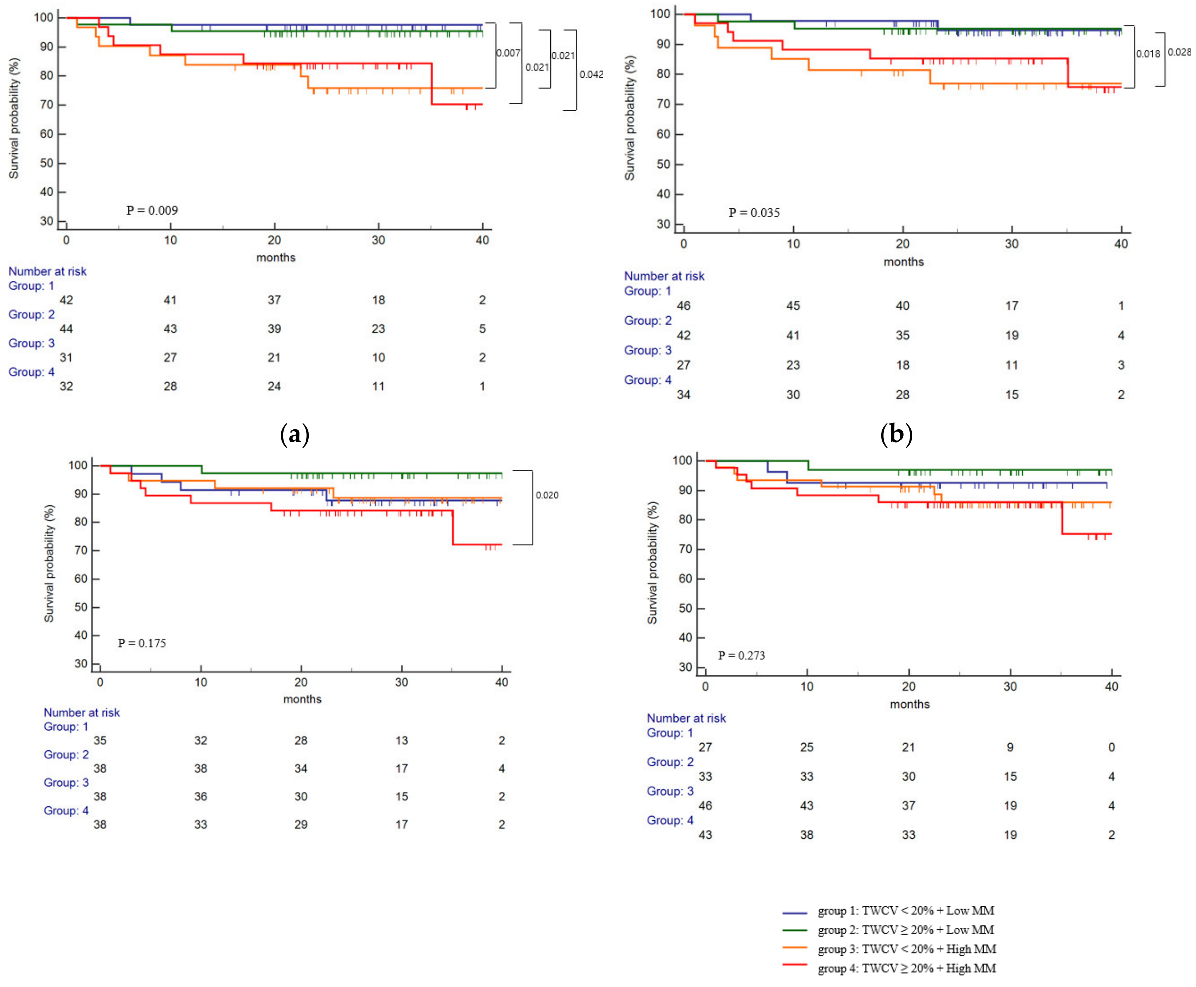

Figure S3. Combined effects of HLA-DQ eplet mismatch and TAC-IPV on the risk of HLA-DQ dnDSA development in class II HLA mismatched recipients. (a) In the single molecular mismatch analysis, higher dnDSA-free survival was observed in group 1 compared to group 3 ($\rho = 0.007$) and group 2 compared to group 4 ($\rho = 0.042$). Worse dnDSA-free survival was observed in group 4 compared to group 1 ($\rho = 0.021$), and in group 3 compared to group 2 ($\rho = 0.021$). (b) In the total eplet mismatch analysis, worse dnDSA-free survival was observed in group 3 compared to group 1 ($\rho = 0.018$), and group 2 ($\rho = 0.028$). (c) In the antibody verified eplet mismatch analysis, lower dnDSA-free survival was observed in group 4 compared to group 2 ($\rho = 0.020$). (d) In the antibody verified single eplet mismatch analysis, there was no statistical significance between groups.

* TAC-IPV, tacrolimus intrapatient variability (TAC-IPV)

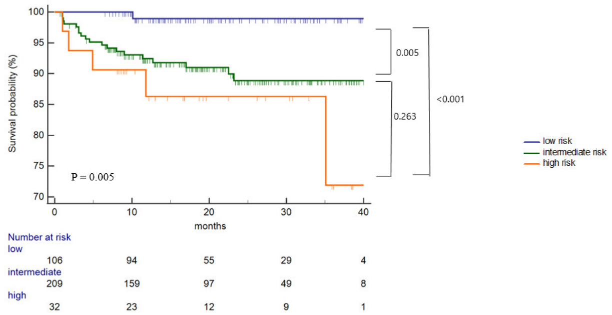

Figure S4. Single molecular eplet mismatch analysis using cut-off from a previous study on the risk of class II dnDSA development. We used different cut-off values for single molecular eplet mismatch risk compared with used in Wiebe et al (13). The high-risk group (HLA-DQ ≥ 15) showed significantly increased risk of dnDSA development compared to the low (HLA-DQ ≤8) ($\rho < 0.001$). There was significant difference between the low and intermediate (HLA-DQ 9-14) risk group (ρ =0.005), but no significant difference was observed between the intermediate and high risk group (ρ =0.263).
