# Supplementary material for: Combined Analysis of HLA Class II Eplet Mismatch and Tacrolimus Levels for the Prediction of De Novo Donor Specific Antibody Development in Kidney Transplant Recipients
Source: Int J Mol Sci. 2022 Jul 1;23(13):7357. doi: 10.3390/ijms23137357 (PMC9267119; doi:10.3390/ijms23137357)
Supplement: Supplementary file 1 [file ijms-23-07357-s001.zip › ijms-1767491-supplementary/Supplementary table.pdf]

Table S1. Validation of proportional hazard assumptions in Multivariate analysis for class II dnDSA development (Schoenfeld test) (n=175)[a].

	Single molecular eplet		Total eplet		Antibody-verified eplet		Antibody-verified single molecular	
	Chi-square	*ρ value*	Chi-square	*ρ value*	Chi-square	*ρ value*	Chi-square	*ρ value*
Eplet MM & TAC-T0								
Age	0.671	0.413	0.563	0.453	0.492	0.483	0.607	0.436
Group	1.744	0.627	0.500	0.919	0.779	0.854	2.519	0.472
Global	2.446	0.654	1.333	0.856	1.376	0.848	3.525	0.474
Eplet MM & TWCV								
Age	0.733	0.392	0.616	0.433	0.548	0.459	0.650	0.420
Group	1.144	0.767	1.898	0.594	0.434	0.933	2.020	0.568
Global	1.793	0.774	2.268	0.687	1.122	0.891	2.850	0.583

[a] Models were adjusted for the following characteristics: age, sex, induction type, and ABO compatibility. Univariate results with *ρ*-value <0.2 were included in the multivariate analysis. Multivariate analysis of each eplet mismatch category was performed separately.

[*] Group 1 represents patients with high TAC-T0 + low/intermediate eplet mismatch, Group 2 represents patients with low TAC-T0 + low/intermediate eplet mismatch, Group 3 represents patients with high TAC-T0 + high eplet mismatch, Group 4 represents patients with low TAC-T0 + high eplet mismatch

* MM, mismatch; TAC-T0, time-weighted tacrolimus trough level; TWCV, TAC-C0 time-weighted coefficient variability

Table S2. Multivariate analysis for HLA-DQ dnDSA development in subgroup (n=149)[a].

	Single molecular eplet		Total eplet		Antibody-verified eplet		Antibody-verified single molecular	
	HR (95% CI)	*ρ value*	HR (95% CI)	*ρ value*	HR (95% CI)	*ρ value*	HR (95% CI)	*ρ value*
Eplet MM & TAC-T0								
Group 1	Reference		Reference		Reference		Reference	
Group 2	1.04 (0.13-8.36)	0.968	1.06 (0.13-8.39)	0.955	4.85 (1.28-18.29)	0.019	3.48 (0.72-16.79)	0.120
Group 3	3.77 (1.41-10.09)	0.008	3.21 (1.18-8.68)	0.022	3.17 (1.18-8.52)	0.022	3.06 (1.13-8.28)	0.028
Group 4	8.03 (2.62-24.66)	0.000	6.22 (2.07-18.66)	0.001	2.92 (0.77-11.09)	0.115	4.09 (1.19-14.09)	0.025
Eplet MM & TWCV								
Group 1	Reference		Reference		Reference		Reference	
Group 2	0.94 (0.19-4.56)	0.939	0.90 (0.19-4.28)	0.899	0.35 (0.04-2.73)	0.315	0.47 (0.06-3.82)	0.482
Group 3	5.17 (1.79-14.85)	0.002	4.56 (1.57-13.26)	0.005	1.47 (0.46-4.74)	0.520	2.17 (0.74-6.35)	0.158
Group 4	4.16 (1.39-12.47)	0.011	3.42 (1.17-9.97)	0.024	2.66 (0.99-7.08)	0.050	2.79 (0.99-7.79)	0.051

[a] Models were adjusted for the following characteristics: age, sex, induction type, and ABO compatibility. Univariate results with *ρ*-value <0.2 were included in the multivariate analysis. Multivariate analysis of each eplet mismatch category was performed separately.

* Group 1 represents patients with high TAC-T0 + low eplet mismatch, Group 2 represents patients with low TAC-T0 + low eplet mismatch, Group 3 represents patients with high TAC-T0 + high eplet mismatch, Group 4 represents patients with low TAC-T0 + high eplet mismatch

* HR, hazard ratio; MM, mismatch; TAC-T0, time-weighted tacrolimus trough level; TWCV, TAC-C0 time-weighted coefficient variability
